# Cardiolipin externalization mediates prion protein (PrP) peptide 106–126-associated mitophagy and mitochondrial dysfunction

**DOI:** 10.3389/fnmol.2023.1163981

**Published:** 2023-06-02

**Authors:** Dongming Yang, Jie Li, Zhiping Li, Mengyang Zhao, Dongdong Wang, Zhixin Sun, Pei Wen, Fengting Gou, Yuexin Dai, Yilan Ji, Wen Li, Deming Zhao, Lifeng Yang

**Affiliations:** National Animal Transmissible Spongiform Encephalopathy Laboratory, College of Veterinary Medicine, State Key Laboratories for Agrobiotechnology, Key Laboratory of Animal Epidemiology of Ministry of Agriculture and Rural Affairs, China Agricultural University, Beijing, China

**Keywords:** mitophagy, autophagy, cardiolipin, prion disease, neurodegenerative disease

## Abstract

Proper mitochondrial performance is imperative for the maintenance of normal neuronal function to prevent the development of neurodegenerative diseases. Persistent accumulation of damaged mitochondria plays a role in prion disease pathogenesis, which involves a chain of events that culminate in the generation of reactive oxygen species and neuronal death. Our previous studies have demonstrated that PINK1/Parkin-mediated mitophagy induced by PrP^106−126^ is defective and leads to an accumulation of damaged mitochondria after PrP^106−126^ treatment. Externalized cardiolipin (CL), a mitochondria-specific phospholipid, has been reported to play a role in mitophagy by directly interacting with LC3II at the outer mitochondrial membrane. The involvement of CL externalization in PrP^106−126^-induced mitophagy and its significance in other physiological processes of N2a cells treated with PrP^106−126^ remain unknown. We demonstrate that the PrP^106−126^ peptide caused a temporal course of mitophagy in N2a cells, which gradually increased and subsequently decreased. A similar trend in CL externalization to the mitochondrial surface was seen, resulting in a gradual decrease in CL content at the cellular level. Inhibition of CL externalization by knockdown of CL synthase, responsible for *de novo* synthesis of CL, or phospholipid scramblase-3 and NDPK-D, responsible for CL translocation to the mitochondrial surface, significantly decreased PrP^106−126^-induced mitophagy in N2a cells. Meanwhile, the inhibition of CL redistribution significantly decreased PINK1 and DRP1 recruitment in PrP^106−126^ treatment but had no significant decrease in Parkin recruitment. Furthermore, the inhibition of CL externalization resulted in impaired oxidative phosphorylation and severe oxidative stress, which led to mitochondrial dysfunction. Our results indicate that CL externalization induced by PrP^106−126^ on N2a cells plays a positive role in the initiation of mitophagy, leading to the stabilization of mitochondrial function.

## Highlights

- PrP^106−126^ caused a temporal course of mitophagy, which gradually increased and subsequently decreased.
- PrP^106−126^ caused a temporal course of CL externalization, which gradually increased and subsequently decreased.
- Inhibition of CL externalization significantly decreased PrP^106−126^-induced mitophagy.
- Inhibition of CL redistribution significantly decreased PINK1 and DRP1 recruitment on mitochondria in PrP^106−126^ treatment but had no significant decrease in Parkin recruitment in mitochondria.
- Inhibition of CL externalization resulted in impaired oxidative phosphorylation and severe oxidative stress, which caused further mitochondrial dysfunction.

## Introduction

Prion diseases are a class of devastating and uniformly fatal neurodegenerative diseases whose pathogen can infect both humans and other animal species, and are based on the aggregation of abnormal protein assemblies that results in a cascade of neurodegenerative pathways (Mead et al., 2019; Maddox et al., 2020). Prion diseases are caused by the accumulation of protease-resistant misfolded form (PrP^Sc^) rich in the beta-sheet structure of cellular prion protein (PrP^C^), which results in astrogliosis and neuronal loss and vacuolation, eventually leading to brain dysfunction and inevitable death (Gu et al., 2002; Minikel et al., 2020; Sevillano et al., 2020). Despite intense studies in recent decades, both the underlying pathogenic mechanism and effective treatment regimen remain unavailable (Takada et al., 2018). The use of PrP^106−126^ in a prion disease model was first reported in 1993 and has gradually been demonstrated to create the best neurotoxic effect (Tagliavini et al., 1991, 1993; Selvaggini et al., 1993; Singh et al., 2002; Forloni et al., 2019). The advantages of PrP^106−126^ are as follows: (1) although PrP^106−126^, such as PrP^Sc^, is highly fibrillogenic, it is soluble in water at micromolar concentrations; (2) this short PrP sequence recapitulates many of the known features of PrP^Sc^ including neurotoxicity, gliotrophic activity, proteinase-K resistance, and a β-sheet structure; and (3) the peptide induces neuronal cell death by way of an apoptotic mechanism, a new paradigm in the field of neurodegeneration (Forloni et al., 2019). Additionally, the effective PrP^106−126^ concentration has also been explored in detail and is reported to be sublethal at 5–50 μM and lethal at 100–200 μM (Tagliavini et al., 1993; White et al., 2001), which our lab has demonstrated for over 20 years.

Healthy mitochondria are imperative for the maintenance of normal neuronal function. Given the non-regenerative nature of neurons, aberrant mitochondrial dynamics and metabolism can cause severe damage to the brain and even the entire nervous system. Numerous studies have uncovered an essential role for mitochondria in the pathogenesis of neurological diseases via different pathways and mechanisms (Kim and Lemasters, 2011; Lou et al., 2020; Lin et al., 2021; Mito et al., 2022). Accordingly, eukaryotic cells, including neural cells, have developed complex mechanisms to eliminate and degrade damaged mitochondria, with the most efficient being mitophagy (Kanki et al., 2009; Harper et al., 2018), which is activated by either ubiquitination or receptor pathways (Palikaras et al., 2018; Pfanner et al., 2019; Zhao et al., 2021). The PINK1/Parkin pathway is the best-characterized ubiquitination-mediated mitophagy mechanism to date; however, defective non-ubiquitination-mediated mitophagy is also recognized to play a role in several neurodegenerative diseases. Cardiolipin (CL), a specific phospholipid found only in the mitochondrial inner membrane, plays a vital and complex role in maintaining mitochondrial homeostasis from fusion and fission to mitophagy and apoptosis (Kagan et al., 2005; Ji et al., 2012; Chu et al., 2013; Liu and Chan, 2017; Ban et al., 2018; Kojima et al., 2019; Falabella et al., 2021). Although the potentially specific role of CL in mitophagy requires further investigation, the discovery of a direct interaction between CL and LC3II uncovered a novel molecular signal that distinguishes CL-mediated mitophagy from ubiquitination-mediated mitophagy (Kagan et al., 2009; Chu et al., 2013, 2014).

A plethora of studies into neurodegeneration have demonstrated that the persistent accumulation of damaged mitochondria in neurons is involved in aging and neurodegenerative disorders, including prion disease (Norat et al., 2020). Our previous study has demonstrated that PrP^106−126^ peptide can cause mitochondrial damage and dysfunction, in turn exacerbating the course of damage induced by PrP^106−126^ based on cellular and animal models (Yang et al., 2008a; Li et al., 2018, 2022; Wu et al., 2019; Zhang et al., 2020). Specifically, mitochondrial homeostasis is gradually disrupted in N2a cells stimulated by PrP^106−126^, causing aggressive fission, reduced fusion, and intensified apoptosis. Furthermore, PrP^106−126^ initiates mitophagy through a PINK1/Parkin-related ubiquitination pathway, which exacerbates mitochondria-mediated apoptosis (Li et al., 2022), indicating that ubiquitination-mediated mitophagy may play a vital role in PrP^106−126^-induced neural damage. Other studies have also suggested that ubiquitination-mediated mitophagy may play a protective role against prion disease or PrP^106−126^ neural damage at the animal level (Lasmézas and Gabizon, 2018; Gao et al., 2020). Although ubiquitination-mediated mitophagy accounts for a large percentage of cellular mitophagic processes, the molecular signals arising from the direct interaction of LC3II with mitophagic factors, such as CL, remain to be elucidated and may provide potential targets for disease treatment.

In the present study, we demonstrate that PrP^106−126^ caused a temporal course of mitophagy, which significantly increased and subsequently decreased using PrP^106−126^ on N2a cells. Moreover, we show that the CL content decreased and that mitophagy was dependent on the externalization of CL to the outer membrane of mitochondria following induction with PrP^106−126^ on N2a cells. Furthermore, we reveal that the suppression of CL-mediated mitophagy resulted in a worse outcome of PrP^106−126^-induced neural damage on N2a cells due to mitochondrial dysfunction. Taken together, our data indicate that mitophagy caused by PrP^106−126^ on N2a cells, and mediated by CL externalization as a marker for the elimination of damaged mitochondria, is beneficial as a result of stabilizing normal mitochondrial function and attenuating neuronal damage.

## Materials and methods

### Cell culture and PrP^106−126^ peptide

Mouse neuroblastoma N2a cells were cultured in Dulbecco's modified Eagle medium (DMEM) (Hyclone, Logan, UT, USA) supplemented with 10% (v/v) fetal bovine serum (Gibco, Grand Island, NY, USA) at 37°C with 5% CO_2_ in a humidified incubator. The PrP^106−126^ peptide was synthesized at >95% purity by Sangon Bio-Tech (Beijing, China) (sequence: KTNMKHMAGAAAAGAVVGGLG), PrP^106−126^ scrambled sequence was: MEVGWYRSPFSRVVHLYRNGK referred from Benoit Schneider's article (Yang et al., 2008b). The peptide was dissolved in 0.1 M phosphate-buffered saline (PBS) to a stock concentration of 1 mM and then shaken at 4°C for 24 h to allow for aggregation. All procedures were performed under sterile conditions, and all experiments were conducted using a final peptide concentration of 150 μM.

### siRNA administration and transfection

The siRNAs targeting murine CL synthase (CLS), phospholipid scramblase-3 (PLS3), and nucleoside diphosphate kinase (NDPK-D), in addition to the negative control siRNA, were synthesized by GenePharm (GENEPHARM). The sequence details are as follows: siCLS: (mouse, 5′-GGGCUACCUGAUUCUUGAATTdTdT-3′, 5′-CCACUCACUUACAUGAUAATTdTdT-3′, 5′-GCUAAGUACUUCAAUCCUUTTdTdT-3′, pooled); siPLS3: (mouse, 5′-GCUGGGAGACCUGUAAUAUTTdTdT-3′, 5′-GGUGAAGACUAAGGAUGAATTdTdT-3′, 5′-CCACGUUCCUCAUCGAUUATTdTdT-3′, pooled); siNDPK-D (mouse, 5′-AGAGCAGCGAACUGUUGAACUdTdT-3′, 5′-GCUCUUAUCAGCUACAUGAGCdTdT-3′, pooled); and siRNA negative control: (mouse, 5′-UUCUCCGAACGUGUCACGUTTACGUGACACGUUCGGAGAATT-3′). The Mito-GFP and DsRed-Mito plasmids were obtained from Clontech (Mountain View, CA USA), and the pSLenti-CMV-mt-mKeima-PGK-Puro-WPRE vector was procured from Obio Technology. Cultured N2a cells were transfected with siRNA or the relative plasmid at a density of 1 × 10^5^ in a 24-well plate using Lipofectamine™ 3000 (Invitrogen, USA) in Opti-MEM (Invitrogen, USA) for 48 h, according to the manufacturer's instructions.

### Mitophagy measured by mt-Keima lentivirus

Mitophagy was directly measured by a fluorescence-based imaging method using mt-Keima, since this novel method was published in *Nature Protocols* in 2017 (Sun et al., 2017) and improved in 2020 (Wang, 2020). Briefly, the mt-Keima lentivirus (also known as the pSLenti-CMV-mt-mKeima-PGK-Puro-WPRE) titers were determined (5 × 10^8^). N2a cells were seeded on 12-well plates and transduced with the mt-Keima virus according to the manufacturer's protocol. After 3 days, green fluorescence was observed in the neurons, indicating successful transfection: green puncta represented normal mitochondria, while red puncta represented mitochondria fused with lysosomes. Data were collected using flow cytometry (BD Calibur). For flow cytometry analysis, harvested cells were washed three times and centrifuged at 0.3 × g for 3 min at 4°C, and resuspended in PBS prior to their analysis on FACSCalibur (BD Bioscience) with 405 and 561 nm lasers and 610/20 filters. Measurement of lysosomal mitochondrially targeted mKeima was made using a dual-excitation ratiometric pH measurement where pH 7 was detected through the excitation at 405 nm and pH 4 at 561 nm. For each sample, 50,000 events were collected and single, Gating for the mKeima treated group, negative control group. Data were analyzed using FlowJo v10.1 (Tree Star).

### Immunoblotting

Extracts for immunoblotting were acquired from N2a cells homogenized in RIPA buffer (radioimmunoprecipitation assay buffer, Thermo Fisher Scientific, Catalog Numbers 89900). Briefly, proteins were separated by electrophoresis and subsequently transferred to the PVDF membrane by electroblotting using standard protocols. The primary antibodies used were anti-LC3II (Cell Signaling Technology, catalog #2775s), anti-COX IV (Proteintech, catalog no. 11242–1-AP), anti-TOM40 (Proteintech, catalog no. 18409–1-AP), anti-MnSOD (Proteintech, catalog no. 24127–1-AP), anti-CLS (Proteintech, catalog no.14845–1-AP), anti-PLS3 (Proteintech, catalog no.28028–1-AP), anti-NDPK-D (Bioss, bs-11902R), anti-PINK1 (Novus Biologicals, catalog no. BC100–494), anti-Parkin (Abcam, catalog no. ab77924), anti-Drp1 (Cell Signaling Technology, catalog #8570), anti-Lamp2 (Cell Signaling Technology, catalog #49067), PGC1a monoclonal antibody (Proteintech, catalog no. 66369–1-Ig), recombinant anti-mtTFA antibody (Abcam, ab252432), GAPDH monoclonal antibody (Proteintech, catalog no. 60004–1-Ig), and anti-Tubulin (Proteintech, catalog no. 11224–1-AP).

### Assessment of CL externalization and content at the mitochondrial surface

Evaluation of CL externalization to the mitochondrial surface was performed using the flow cytometry annexin V-binding assay. Briefly, as previously described (Chu et al., 2013; Kagan et al., 2016; Chao et al., 2019), MitoTracker Red CMXRos (M7512, Life Technologies) was applied to mark mitochondria before harvesting, and isolated crude mitochondria were subsequently incubated with FITC-labeled annexin V to stain surface-exposed CL prior to immediate analysis by flow cytometry (BD Calibur). The FITC fluorescence from gated red fluorescent mitochondria (MitoTracker Red) was measured to evaluate the binding of annexin V to mitochondria. Data are presented as the relative FITC fluorescence intensity in comparison with that of mitochondria isolated from control samples. The dye 10-N-nonyl acridine orange (NAO), which specifically binds with high affinity to cardiolipin but not to zwitterionic or other anionic phospholipids, was used to assess the mitochondrial cardiolipin content in living cells (Mileykovskaya et al., 2001; Garcia Fernandez et al., 2004; Kaewsuya et al., 2007). Briefly, cultured cells or isolated mitochondria were exposed to 1 μM NAO for 15–20 min in high-glucose DMEM and subsequently washed three times with PBS. Here, two methods were used to detect NAO fluorescence intensity to overcome method drawbacks: (1) confocal microscopy (Nikon, Tokyo) as described below (Kirkland et al., 2002), with NAO excitation at 488 nm; and (2) measurement using a GloMax^®^ 96 Microplate Luminometer at 485/530 nm (Hsu et al., 2015).

### Confocal microscopy

N2a cells were cultured on 24-well coverslips and subsequently transfected with Mito-GFP, DsRed-Mito, Thioflavin T, or siRNA before treatment with PrP^106−126^ or the control peptide. The specific processes were carried out in accordance with our previous studies (Song et al., 2022). Cells were washed twice with PBS before being fixed with 4% paraformaldehyde for 30 min. After washing twice with PBS, the cells were treated with immunostaining permeabilization buffer containing X-100 (Beyotime biotechnology, P0096) for 5–10 min at room temperature to permeabilize them. The cells were then blocked using an immunostaining blocking buffer (Beyotime Biotechnology, P0102) for 1 h at room temperature, followed by overnight incubation with specific primary antibodies (as described in the immunoblotting section) at 4°C (West et al., 2015). Following the PBS rinsing step, the cells were incubated with secondary antibodies for 1 h at 37°C and then washed with PBS five times for 5 min each. The coverslips were mounted on microscope glass slides using a fluorescent antifading buffer (Bioworld Technology, BD5014). Images were acquired using a Nikon A1HD25 confocal microscope at a magnification of 100 × (oil immersion lens). Approximately 10–15 unique images were captured at random for each sample. The acquired images were quantified using ImageJ software.

### Tunel and cell viability assay

N2a cells were seeded into a 24-well plate before treatment with peptide and then used with The One Step TUNEL Apoptosis Assay Kit (Beyotime, catalog no. C1086) in accordance with the manufacturer's instructions before observation using A1 confocal microscope (Nikon). N2a cells' viability in response to PrP^106−126^ was measured using Cell Counting Kit-8 (Beyotime). After treatment, the CCK-8 solution was directly added to the cell culture medium and incubated for 30 min at 37°C and 5% CO2. The absorbance of the samples was measured using a microplate reader (Thermo Fisher Scientific, USA) at 450 nm. The cell viability was expressed as a percent of the untreated control.

### Transmission electron microscopy

To collect samples for microscopic, N2a cells were harvested to do further certain processes after PrP^106−126^ infections. Cell samples were cut into small pieces after fixation in 5% glutaraldehyde in 0.1-M sodium cacodylate buffer (pH 7.4) for 4 h at 4°C and then fixed in glutaraldehyde were rinsed with sodium cacodylate buffer and then further fixed in 1% OsO4 in 0.1 M sodium cacodylate buffer on ice for 2 h before dehydration with acetone. After dehydrating with a series of ethanol and acetone, the cell pellets were embedded in resin before polymerization at 60°C for 48 h. Ultrathin sections (70 nm) were mounted onto copper grids and counter-stained with 4% uranyl acetate and lead citrate, and images were finally observed under a transmission electron microscope (HITACHI HT7700, Japan) operating at 120 kV.

### Mitochondrial isolation

Mitochondrial and cytoplasmic proteins were extracted from N2a cells using a Mitochondria Isolation Kit for Cultured Cells (89874, Thermo Fisher Scientific). Briefly, cells were harvested and subsequently washed three times with 0.1 M PBS, homogenized in lysis buffer containing protease inhibitors, and centrifuged at different speeds. The cytosolic fraction was collected after centrifugation at 3,000 × g for 15 min, and isolated mitochondria were recovered after further centrifugation at 12,000 × g for 5 min. All procedures were carried out at 4°C following the manufacturer's instructions.

### mtDNA content and gene expression and measurement of mitochondrial function

Total DNA was extracted from N2a cells and quantitated by spectrophotometry (NanoDrop 2000). Real-time PCR was performed using the ViiA7 Fast Real-Time PCR System (ABI) and SYBR Green Master Mix (Bio-Rad). Primer sequences are as follows: mtDNA (F: 5′-CCTATCACCCTTGCCATCAT-3′ and R: 5′-GAGGCTGTTGCTTGTGTGAC-3′) and genomic DNA (gDNA) (F: 5′-ATGGAAAGCCTGCCATCATG-3′ and R: 5′-TCCTTGTTGTTCAGCATCAC-3′). Data are expressed as the fold change relative to control samples, calculated using the comparative CT method (2^−Δ*ΔCT*^).

Gene of *PGC-1*α and *mTfam* expression was also measured by real-time PCR. Total cellular RNA was extracted from N2a cells after temporal treatments with PrP^106−126^ by using a total RNA Extraction kit (Aidlab Biotechnologies, Beijing, China) according to the manufacturer's instructions. The cDNA was then generated using HiScript III First Strand cDNA Synthesis Kit (+gDNA wiper) (Vazyme, Biotech Co. Ltd). Real-time PCR was performed with the cDNA using the ViiA7 Fast Real-Time PCR System (ABI) and SYBR Green Master Mix (Bio-Rad). Primer sequences are as follows mouse *PGC1*α: (F: 5′-ACAACGCGGACAGAATTGAG-3′ and R: 5′-GTTTCGTTCGACCTGCGTAA-3′), mouse *mTfam:* (F: 5′-AAGGATGATTCGGCTCAGG-3′ and R: 5′-GGCTTTGAGACCTAACTGG-3′), and mouse *Tubulin* (F: 5′-ACATGGCTTGCTGCCTATTG-3′ and R: 5′-GACCACAGTGAGGCTGGTAA-3′) as a control due to its stable internal expression. Each cDNA sample was performed for three biological triplicates, and Ct values of each replicate were normalized to Tubulin cDNA using the 2 ^−Δ*ΔCt*^ method. For relative expression (fold), internal control samples were centered at 1.

Reactive oxygen species (ROS) in N2a cells were measured using 2′,7′-dichlorodihydrofluorescein diacetate (Beyotime, Shanghai, China). Mitochondrial superoxide was detected using the MitoSOX™ Red mitochondrial superoxide indicator (M36008, Thermo Fisher Scientific). The mitochondrial membrane potential (MMP) was evaluated using a JC-1 Mitochondrial Membrane Potential Assay Kit (Beyotime, Shanghai, China). ATP was measured using an ATP Determination Kit (Beyotime, Shanghai, China). The mitochondrial complex activity was measured using a Micro Mitochondrial Respiratory Chain Complex Activity Assay Kit (Solarbio, China). Luminescence was measured using a GloMax^®^ 96 Microplate Luminometer, and fluorescence signals were analyzed using a BD Calibur Fluorescence-activated Cell Sorting Instrument. All processes were carried out following the manufacturer's instructions.

### Thioflavin T assay

Thioflavin T is a fluorescent stain that specifically binds to beta-sheet-rich structures, which was used to detect the misfolded PrP protein. Upon binding, the dye exhibits a red-shift in its emission spectrum and increased fluorescence intensity. Briefly, the following protocol was below and referred from two articles (Florio et al., 2003; Chen et al., 2020). A total of 1 mM stock solution of Thioflavin T (Th T, MCE: HY-D0218) was prepared in deionized water and filtered through a 0.2 μm syringe filter before use. Thioflavin T was then diluted in PBS buffer (pH 7.4) to a final concentration of 25 μM. For N2a cells, they were seeded into a Lumox 96 multiwell black plate and treated with 150 μM PrP^106−126^ for 24 h. The cells were washed three times with PBS before Thioflavin T was added to each well (100 μL volume) of the plate. Alternatively, for preformed PrP^106−126^ fibrils, Thioflavin T was diluted in PBS buffer (pH 7.4) to a final concentration of 25 μM in each well (100 μL volume) of a Lumox 96 multiwell plate. A total of 150 μM of preformed PrP^106−126^ fibrils were added to the appropriate wells, and the contents were mixed by pipetting. The plate was sealed and placed in a shaking incubator (600 rpm) at 37°C. The data were analyzed by a GloMax^®^ 96 Microplate Luminometer at 450/480 nm or confocal.

### Statistical analysis

All assays were repeated three times, and the number of replicates is presented by individual data points on each graph. Data are reported as the mean ± SD. All comparisons of parametric data were performed using one-way ANOVA followed by Tukey's *post hoc* test and an unpaired *t-*test (two-tailed) in GraphPad Prism version 7.0 (La Jolla, CA, USA). The gray level was analyzed using ImageJ (National Institutes of Health, Bethesda, MD, USA). A *P*-value of < 0.05 was considered statistically significant.

## Results

### PrP^106−126^ elicits a temporal course of mitophagy in N2a cells

**Figure 1:**
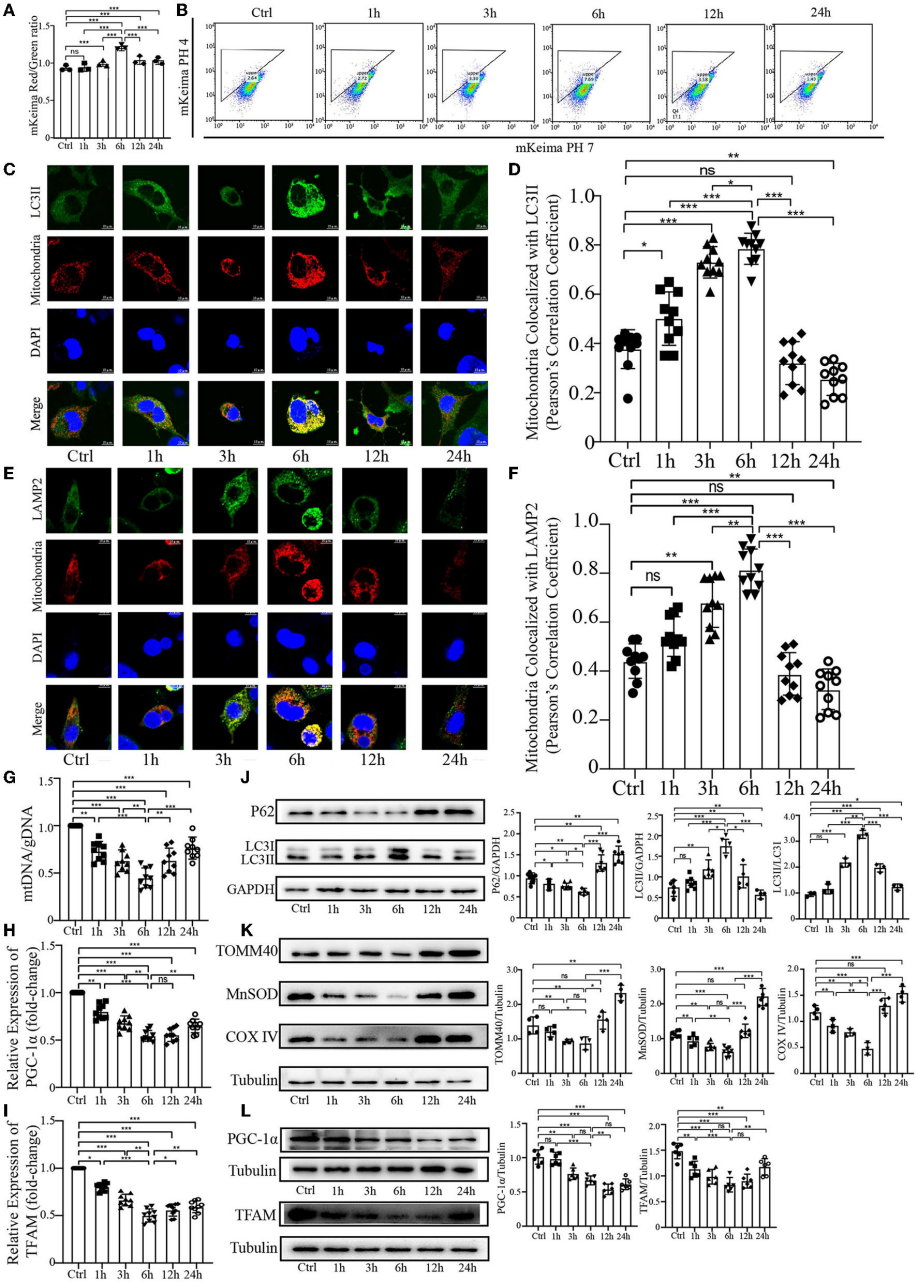
Temporal course of mitophagy after the addition of 150 μM PrP^106−126^ to N2A cells. **(A, B)** The temporal course of mitophagy induced by PrP^106−126^ was characterized by changes in the mKeima fluorescence ratio. **(C–F)** Detection of the temporal course of mitophagy after PrP^106−126^ stimulated on N2a cells using immunofluorescence colocalization of the mitochondrial marker DsRed-Mito and LC3II or LAMP2. Scale bar: 10 μm. **(G)** The temporal mtDNA/gDNA ratio was assessed by real-time PCR, reflecting the relative number of mitochondria after PrP^106−126^ was stimulated on N2a cells. **(H, I)** The messenger RNA relative expression level of PGC-1α and mitochondrial transcription factor A (TFAM) were detected at temporal time-course after PrP^106−126^ treatments using quantitative real-time polymerase chain reaction. **(J, K)** Immunoblotting of temporal mitochondrial and autophagic markers obtained from the N2a cells treated with PrP^106−126^. **(L)** Immunoblotting of temporal mitochondrial biosynthesis-related protein expression levels (PGC-1α and TFAM) obtained from N2a cells with PrP^106−126^ treatment. Tubulin and GAPDH were used as loading controls. Data are presented as the mean ± SD; ns, not significant; *P < 0.05; **P < 0.01; ***P < 0.001. All experiments were repeated at least three times and PrP^106−126^ treatments were a temporal course from 0 to 24 h. Statistical analysis was done using a one-way analysis of variance with Tukey post-tests and unpaired *t-*test (two-tailed).

Our previous study demonstrated that PrP^106−126^ peptide can cause mitophagy deficiency in a cellular model during the late stage of PrP^106−126^ treatment. To further explore the effect of PrP^106−126^ peptide on host mitophagy, we decided to use 150 μM PrP^106−126^ in N2a cells to fully observe the character of mitophagy induced by PrP^106−126^ peptide, since 100 μM PrP^106−126^ may not significantly induce mitophagy during the early stages of PrP^106−126^ treatment (Li et al., 2022). Before the experiments, we first detected the cell viability, toxicity data. and the state of 150 μM PrP^106−126^ on N2a cells (Supplementary material). We took advantage of the coral-derived protein Keima, since the fluorescence of mitochondrial matrix-targeted Keima (COX8-mKeima) changes from green to red within the acidic lysosome (pH 4.0) after mitophagy (Sun et al., 2017). We also evaluated mitophagy by observing the changes in colocalization between the mitochondrial Marker DsRed-Mito and the autophagosome protein LC3II and LAMP2. As shown in Figures 1A, B (mKeima), a gradual increase in mitophagy was observed following PrP^106−126^ treatment, which reached a peak at 6 h and then slowly decreased until 24 h. Moreover, we observed a similar trend in the colocalization of GFP-LC3II and LAMP2 with mitochondria by confocal microscopy (Figures 1C–F). PrP^106−126^ treatment enhanced the colocalization of GFP-LC3II-positive puncta and MitoTracker Red, indicating an increase in the number of mitophagosomes. In combination with immunoblotting of LC3 (LC3II) and P62 in the isolated mitochondrial samples, the autophagic trend gradually increased until 6 h and subsequently decreased until 24 h, which is consistent with the general autophagic trend (Figure 1J). Furthermore, we used the ratio of mitochondrial DNA (mtDNA) to genomic DNA (gDNA) to quantitate the mitochondrial level at six timepoints between 1 h and 24 h after treatment with PrP^106−126^ peptide (Figure 1G). The mtDNA/gDNA ratio exhibited the same trend in the peptide-treated group vs. the control, reaching a trough at 6 h. Combined with the mRNA expression level of PGC-1α and TFAM after the temporal treatment of PrP^106−126^ (Figures 1H, I), we found the mRNA expression level of PGC-1α and TFAM was also significantly decreased on whole time points and the lowest level was at 6 h after PrP^106−126^ temporal treatments. Meanwhile, we also detected the mitochondrial biosynthesis-related proteins (PGC-1α and TFAM) to determine the breakdown of mitochondrial biosynthesis level (Figure 1L). We found that both two protein levels were gradually significantly decreased after temporal treatments of PrP peptide. These results show that PrP^106−126^ causes mitochondrial biosynthesis breakdown and decreased. Additionally, the use of different levels of mitochondrial marker proteins mirrored the mitochondrial dynamics. The levels of TOM40 in the outer mitochondrial membrane, MnSOD in the matrix, and COXIV in the inner mitochondrial membrane were decreased following PrP^106−126^ treatment until reaching a trough at 6 h, before subsequently increasing until 24 h (Figure 1K). Overall, these data demonstrate in detail the dynamic trend in mitophagy and mitochondrial biosynthesis induced by PrP^106−126^ peptide in N2a cells.

### CL externalizes to the mitochondrial outer membrane and decreases its content in response to PrP^106−126^

**Figure 2:**
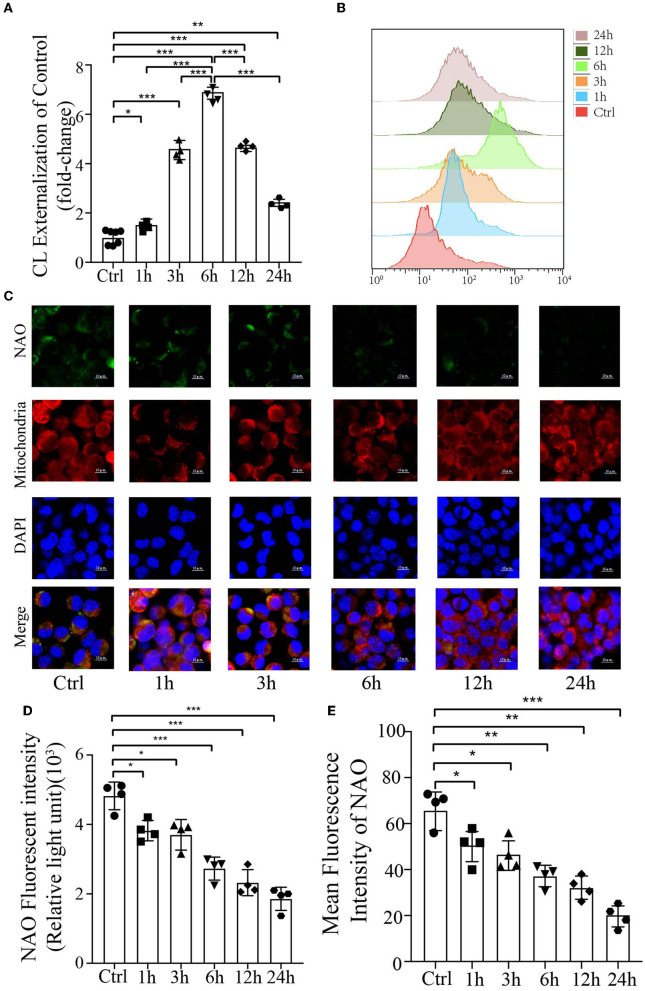
Analysis of mitochondrial CL externalization and content after the addition of 150 μM PrP^106−126^ to N2A cells. **(A, B)** The temporal exposure of CL on the mitochondrial surface was detected by Annexin V. **(C, E)** The temporal course of CL content induced by PrP^106−126^ stimulated on N2a cells was assessed by NAO staining as detected by confocal microscopy. Scale bar: 10 μm. **(D)** Relative CL level was assessed by NAO staining as detected by a microplate luminometer. NAO, 10-N-nonyl acridine orange. Data are presented as the mean ± SD; ns, not significant; *P < 0.05; **P < 0.01; ***P < 0.001. All experiments were repeated at least three times, and PrP^106−126^ treatments were a temporal course from 0 to 24 h. Statistical analysis was done using one-way analysis of variance with Tukey post-tests and unpaired *t*-test (two-tailed).

Cardiolipin is a mitochondrial-specific phospholipid that is only distributed in the inner membrane but has been reported to be redistributed under several disease conditions (Chu et al., 2014; Hsu et al., 2015; Song et al., 2019); however, no studies have demonstrated its externalization or content in prion disease or model of prion disease. To examine the mitochondrial redistribution and content of CL during mitophagy induced by PrP^106−126^ peptide, the annexin V-FITC externalization assay was used to monitor the externalization of CL to the mitochondrial surface. Stimulation with PrP^106−126^ caused robust externalization of CL, which peaked at 6 h and then gradually decreased (Figures 2A, B). Moreover, the CL-specific dye NAO was utilized to monitor CL levels in live cells, directly measuring its luminescence using a microplate luminometer, in addition to monitoring its mean fluorescence intensity by confocal microscopy. Both these results indicate that the CL content decreased dramatically from 1 h to 24 h following PrP^106−126^ stimulation (Figures 2C–E). In comparison with the control group, the PrP^106−126^ peptide-treated group showed a marked decrease in NAO fluorescence intensity at 6 h, with greater than a 2-fold decrease seen at 24 h. Furthermore, confocal data demonstrate that the CL content in the PrP^106−126^ peptide-treated group was significantly decreased at 1 h and very significantly reduced at 6 h (Figures 2C, E). Therefore, PrP^106−126^ treatment caused CL externalization to the outer mitochondrial membrane and a significant decrease in its content. Despite the continuous decrease in the CL content, a peak in CL externalization was reached at 6 h following PrP^106−126^ peptide treatment, which then subsequently decreased.

### CL externalization induced by PrP^106−126^ prompts mitophagy via the recruitment of LC3II

**Figure 3:**
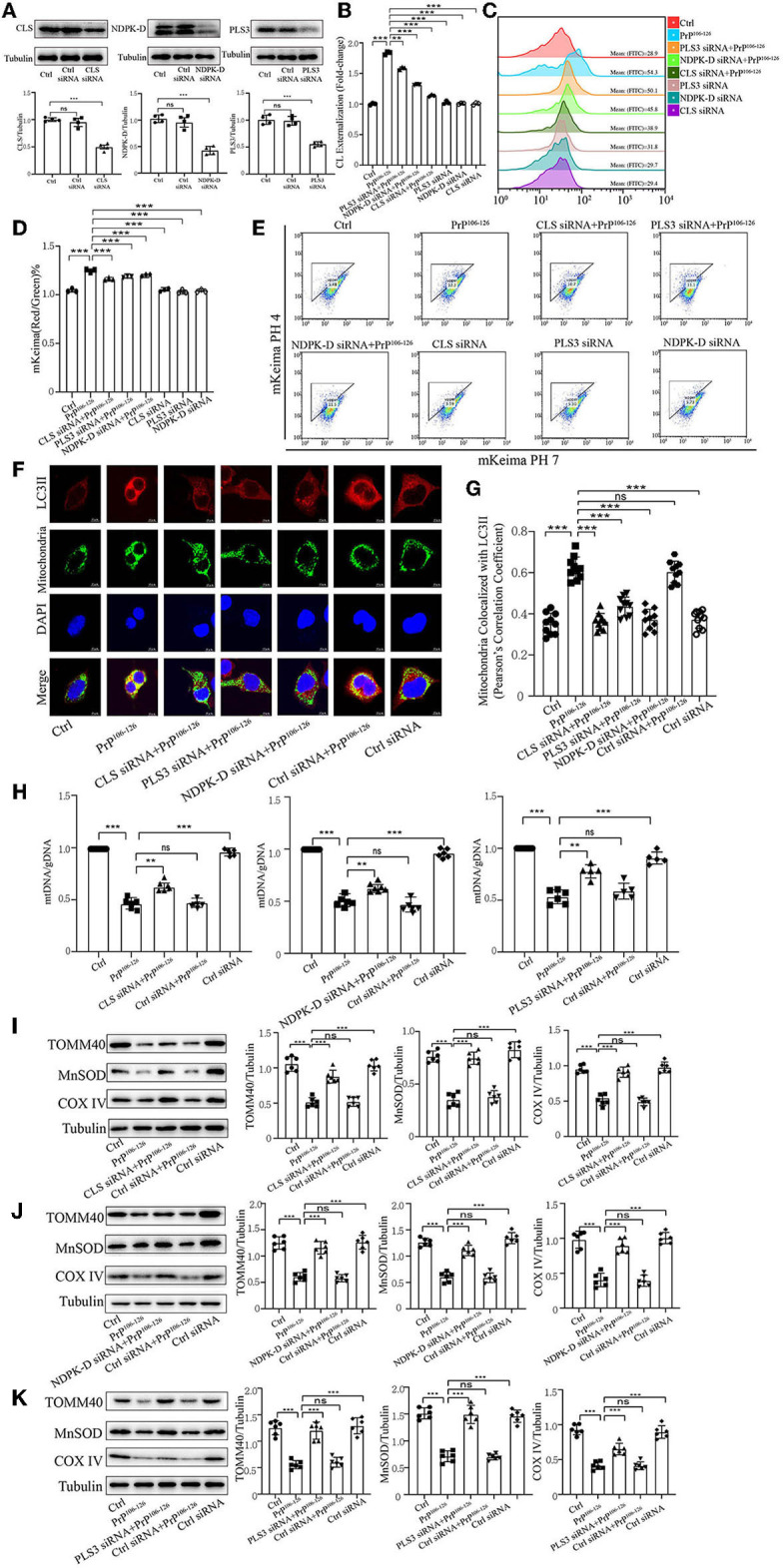
Inhibition of CL externalization dampens 150 μM PrP^106−126^-induced mitophagy in N2A cells. **(A)** Immunoblotting of PLS3, CLS, and NDPK-D levels after knockdown. **(B, C)** Exposure of CL on the mitochondrial surface was detected by Annexin V after the inhibition of CL externalization. **(D, E)** Detection of mitophagy induced by PrP^106−126^ was characterized by changes in the mKeima fluorescence ratio after the inhibition of CL externalization. **(F, G)** Detection of immunofluorescence colocalization of LC3II and the mitochondrial marker mito-GFP after the inhibition of CL externalization after PrP^106−126^ stimulated on N2a cells. Scale bar: 10 μm. **(H)** The mtDNA/gDNA ratio was assessed by real-time PCR, reflecting the relative number of mitochondria after the inhibition of CL externalization. **(I–K)** Immunoblotting of mitochondrial markers obtained from the N2a cells treated with PrP^106−126^ after the inhibition of CL externalization. Tubulin was used as the loading control. Data are presented as the mean ± SD; ns, not significant; *P < 0.05; **P < 0.01; ***P < 0.001. All experiments were repeated at least three times and PrP^106−126^ treatment was 6 h, causing a peak in mitophagy. Statistical analysis was done using one-way analysis of variance with Tukey post-tests and unpaired *t*-test (two-tailed).

Previous research has uncovered that CL redistribution to the surface of mitochondria acts as a mitophagic signal by specifically interacting with the autophagic protein LC3II (Chu et al., 2013, 2014; Kagan et al., 2014). Based on our previous discovery that CL externalization is induced by PrP^106−126^ treatment, we speculated that CL externalization is involved in mitophagy in the cellular model of prion disease. To investigate two essential enzymes that catalyze different stages of CL translocation from the inner to the outer mitochondrial membrane (Figure 3A), we knocked down PLS3 and NDPK-D *in vitro*. Knockdown of these two proteins using siRNA reduced the exposure of CL to the outer mitochondrial membrane following PrP^106−126^ treatment (Figures 3B, C). Additionally, monitoring of mKeima by flow cytometry revealed that PrP^106−126^-induced mitophagy was decreased after the knockdown of these two enzymes (Figures 3D, E). Moreover, the ratio of mtDNA/gDNA, which was used to quantitate mitochondrial levels, indicates that the number of mitochondria decreased following the addition of PrP^106−126^ (Figure 3H). GFP-LC3II colocalization with mitochondria was increased (Figures 3F, G), and the loss of mitochondrial proteins was inhibited (Figures 3J, K) following the addition of PrP^106−126^. To confirm the role of CL in recognizing injured mitochondria during PrP^106−126^-induced mitophagy, we knocked down CL synthase *in vitro* using siRNA (Figure 3A). After injury with PrP^106−126^, the level of CL externalization to the outer mitochondrial membrane was diminished (Figures 3B, C). Moreover, flow cytometry of mKeima, in combination with the mtDNA/gDNA ratio, demonstrates that PrP^106−126^-induced mitophagy was decreased after CLS knockdown (Figures 3D, E, H). Furthermore, colocalization of GFP-LC3II puncta with mitochondria and loss of mitochondrial proteins were reduced following the addition of PrP^106−126^ (Figures 3F, G, I). Therefore, decreased externalization of CL due to the knockdown of PLS3, NDPK-D, and CLS significantly decreased mitophagy by reducing LC3II recruitment to mitochondria, exacerbating the accumulation of damaged mitochondria.

### Decreased CL repartition following PrP^106−126^ treatment leads to a significant reduction in PINK1 recruitment but has a slight effect on Parkin

**Figure 4:**
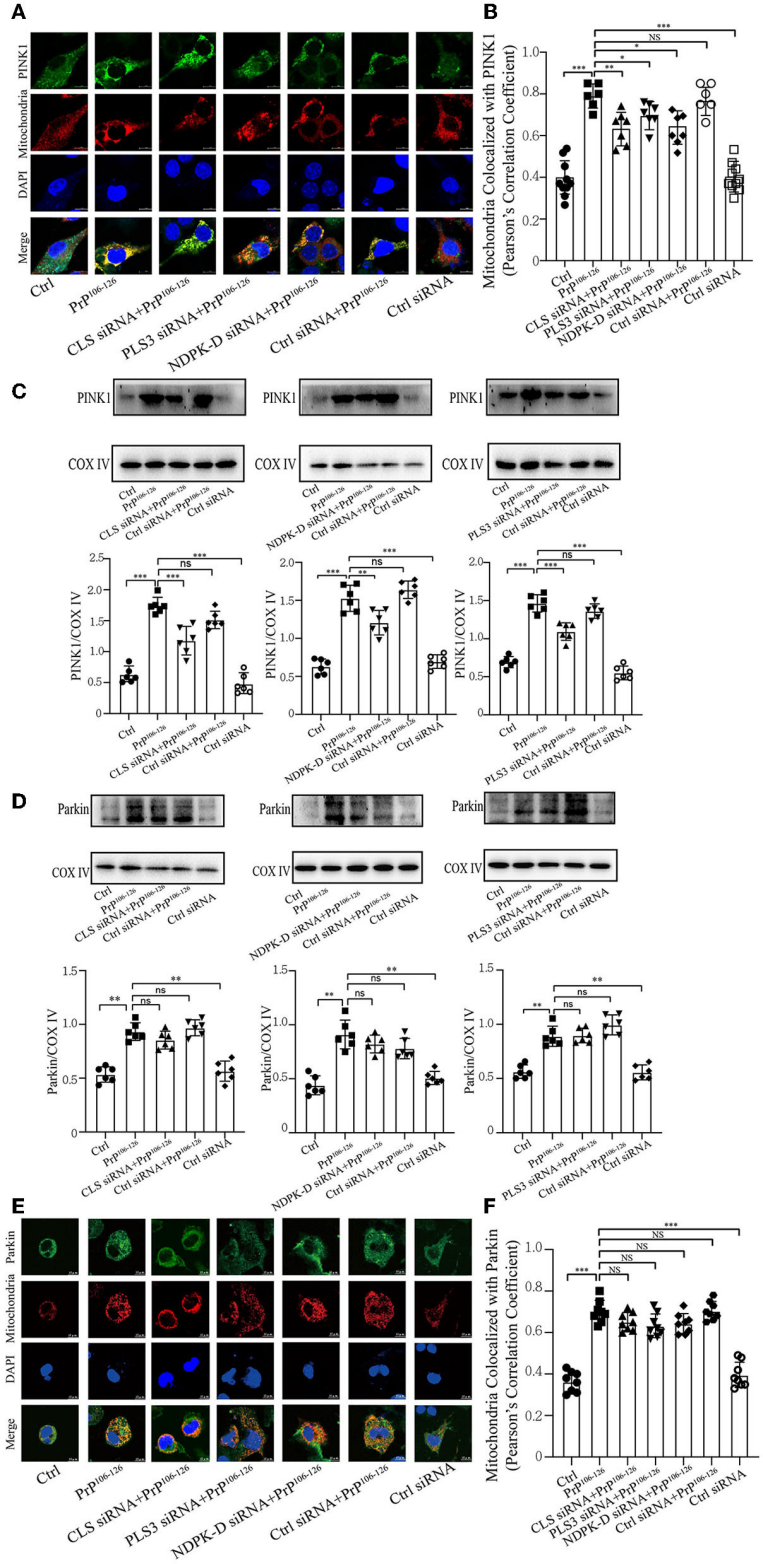
CL repartition during 150 μM PrP^106−126^ treatment in N2A cells leads to a reduction in mitochondrial PINK1 without significantly affecting Parkin levels. **(A, B, E, F)** Detection of immunofluorescence colocalization of the mitochondrial marker DsRed-Mito and PINK1 or Parkin after the inhibition of CL externalization on N2a cells treated with PrP^106−126^. Scale bar: 10 μm. **(C, D)** Immunoblotting of PINK1 and Parkin levels recruiting on mitochondria after the inhibition of CL externalization with PrP^106−126^ treatment using isolated mitochondrial samples. Cox IV, mitochondrial inner membrane protein, was used as the loading control. Data are presented as the mean ± SD; ns, not significant; *P < 0.05; **P < 0.01; ***P < 0.001. All experiments were repeated at least three times and PrP^106−126^ treatment was 6 h, causing a peak in mitophagy. Statistical analysis was done using one-way analysis of variance with Tukey post-tests and unpaired *t*-test (two-tailed).

Phosphorylation of PARK2/Parkin by PTEN-induced putative kinase 1 (PINK1) plays an essential role in ubiquitin-mediated mitophagy (Geisler et al., 2010a; Lazarou et al., 2015; Gan et al., 2022). Additionally, our previous study has demonstrated that PrP^106−126^ treatment causes a deficiency in PINK1/Parkin-mediated mitophagy, which aggravates neural apoptosis on the N2a cellular model (Li et al., 2022). Another study has highlighted that CL promotes electron transport between ubiquinone and complex I to rescue PINK1 deficiency (Vos et al., 2017); however, the effect of CL externalization on PARK2/Parkin remains unknown. Therefore, we investigated whether the defective CL externalization influences the levels of PINK1 or Parkin recruitment to mitochondria, since depolarized mitochondria induced by PrP^106−126^ peptide are recognized via a PINK1/Parkin-dependent mechanism. Confocal microscopy and immunoblotting (using isolated mitochondrial samples) demonstrate that the colocalization of PINK1 with mitochondria was significantly reduced after CL repartition, and the levels of PINK1 were also significantly decreased (Figures 4A–C). However, the levels of Parkin recruited to mitochondria were not significantly decreased after CL repartition using both isolated mitochondrial samples (Figure 4D) and confocal microscopy (Figure 4E). Taken together, these results suggest that inhibition on CL repartition induced by PrP^106−126^ significantly reduces PINK1 recruitment to mitochondria but has no significantly reduced effect on Parkin recruitment.

### Deficient CL externalization following PrP^106−126^ treatment downregulates DRP1 translocation to mitochondria

**Figure 5:**
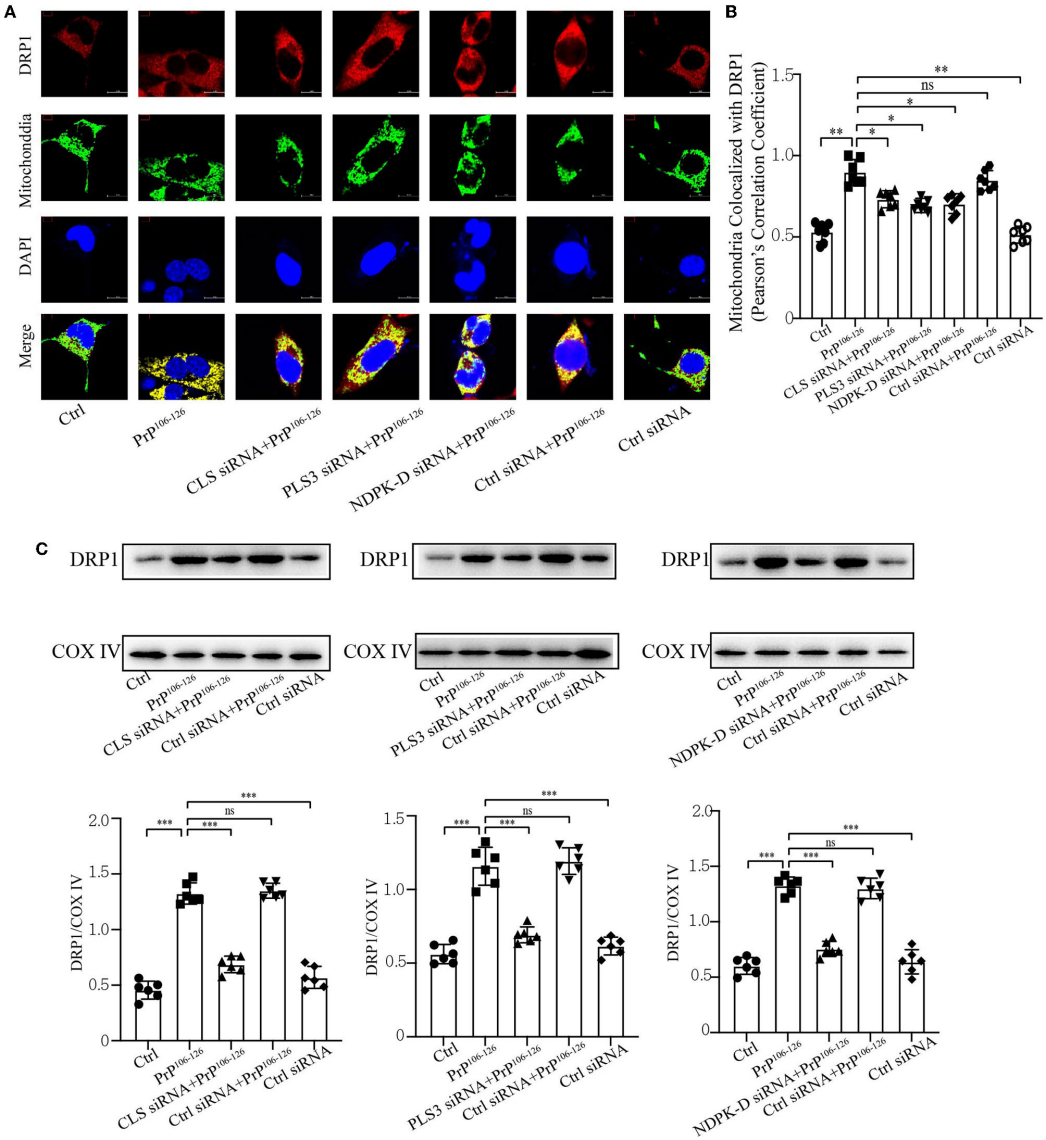
Inhibition of CL externalization downregulates DRP1 translocation to mitochondria during the early stage of mitophagy after 150 μM PrP^106−126^ treatment in N2A cells. **(A, B)** Detection of immunofluorescence colocalization of the mitochondrial marker DsRed-Mito and DRP1 after the inhibition of CL externalization on N2a cells treated with PrP^106−126^. Scale bar: 10 μm. **(C)** Immunoblotting of DRP1 levels recruiting on mitochondria after the inhibition of CL externalization with PrP^106−126^ treatment using isolated mitochondrial samples. Cox IV, mitochondrial inner membrane protein, was used as the loading control. Data are presented as the mean ± SD; ns, not significant; *P < 0.05; **P < 0.01; ***P < 0.001. All experiments were repeated at least three times and PrP^106−126^ treatment was 6 h, causing a peak in mitophagy. Statistical analysis was done using one-way analysis of variance with Tukey post-tests and unpaired *t*-test (two-tailed).

Previous studies have shown that CL externalization to the mitochondrial surface is essential for the activation of fission by directly interacting with DRP1 to enhance its GTPase activity (Ugarte-Uribe et al., 2014; Francy et al., 2017; Gokul, 2018; Rosdah et al., 2020; Mahajan et al., 2021). Moreover, mitochondrial fragmentation plays a vital role in mitophagic processes, in which DRP1 acts as the main regulator of mitochondrial fission and subsequent elimination by autophagy (Twig et al., 2008; Zhang et al., 2013; Lin et al., 2020; Oshima et al., 2021). Our previous study demonstrated that the onset of PrP^106−126^ stimulation is closely related to DRP1-dependent mitochondrial fragmentation (Li et al., 2018), which plays a pivotal role in PrP^106−126^-associated mitochondrial dysfunction and neuronal apoptosis. Accordingly, we speculated that deficiency in CL externalization influences DRP1 translocation to mitochondria. Confocal microscopy shows that PrP^106−126^-induced deficiency in CL externalization significantly decreased DRP1 translocation to mitochondria (Figures 5A, B). Moreover, immunoblotting, using isolated mitochondrial samples, demonstrates that deficient CL redistribution following PrP^106−126^ treatment reduced DRP1 levels in mitochondria by using mitochondrial samples (Figure 5C). Thus, these results suggest that the inhibition of CL externalization reduces DRP1 recruitment to mitochondria, potentially decreasing mitochondrial fission and inhibiting mitophagy.

### CL externalization ameliorates PrP^106−126^-induced oxidative stress and mitochondrial dysfunction

**Figure 6:**
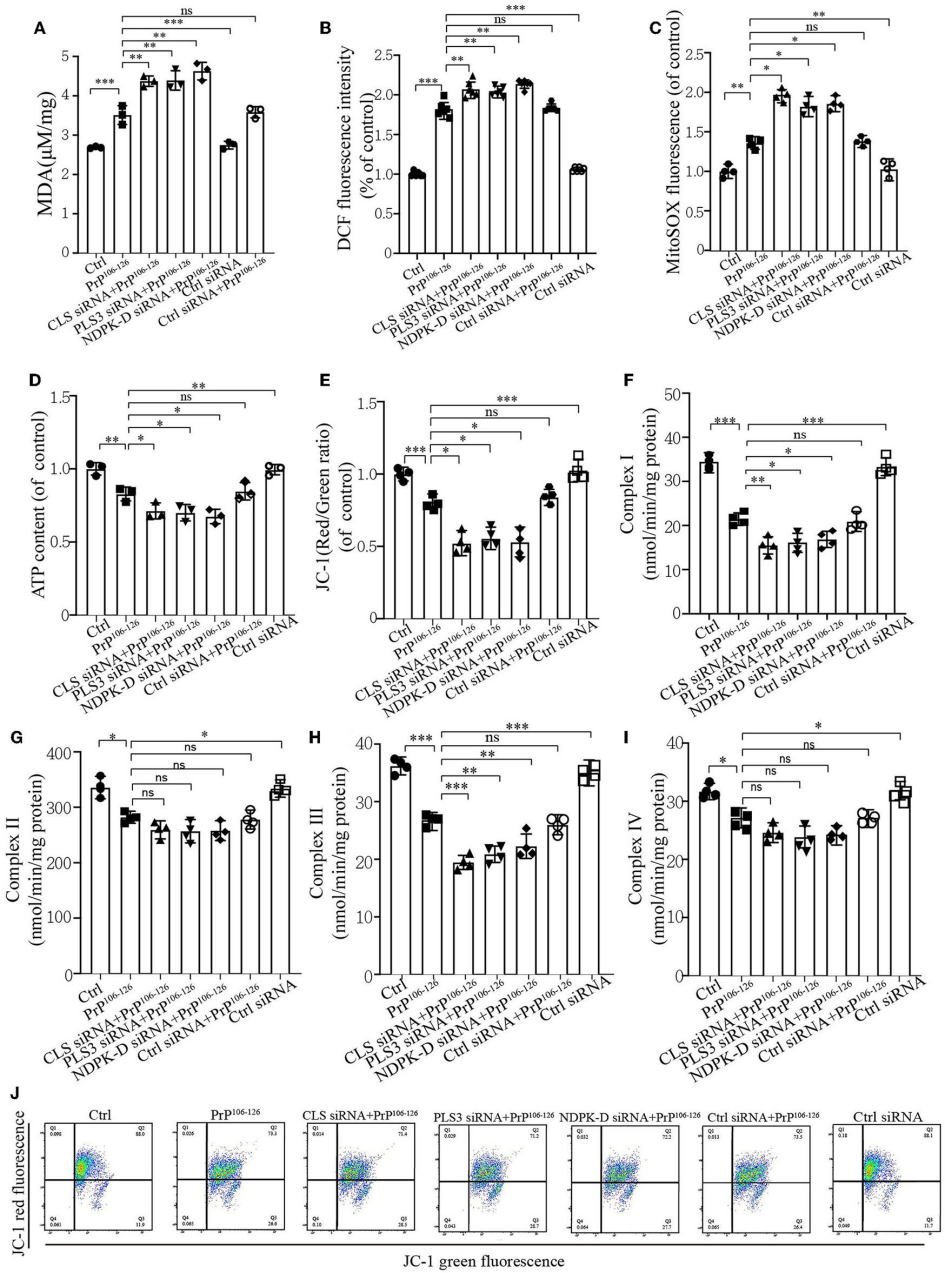
CL externalization ameliorates 150 μM PrP^106−126^-induced oxidative stress and mitochondrial dysfunction in N2a cells. **(A)** Malonaldehyde (MDA) levels after the inhibition of CL externalization were detected using a microplate luminometer. **(B)** ROS production after the inhibition of CL externalization was detected using a microplate luminometer. **(C)** Mitochondrial superoxide after the inhibition of CL externalization was detected using a microplate luminometer. **(D)** ATP levels after the inhibition of CL externalization were detected using a microplate luminometer. **(E, J)** Fluorescence was detected by flow cytometry (FACS) analysis of JC-1 as a marker of mitochondrial membrane potential (MMP) after the inhibition of CL externalization. **(F–I)** Mitochondrial respiratory chain complexes after the inhibition of CL externalization were detected using a microplate luminometer. Data are presented as the mean ± SD; ns, not significant; *P < 0.05; **P < 0.01; ***P < 0.001. All experiments were repeated at least three times, and PrP^106−126^ treatment was 6 h, causing a peak in mitophagy. Statistical analysis was done using one-way analysis of variance with Tukey post-tests and unpaired *t*-test (two-tailed).

Mitophagy caused by PrP^106−126^ is required for mitochondrial quality control (Keller et al., 2019). As we found that CL externalization increased the mitophagy level to clean damaged mitochondria induced by PrP^106−126^ and had a positive effect on PINK1 and DRP1 recruitment, the uncertain effects of CL externalization induced by PrP^106−126^ prompted us to investigate the consequences of oxidative stress on mitochondrial dysfunction, which is widely reported in Prion disease (Milhavet et al., 2000). The results show that the suppression of CL externalization after PrP^106−126^ stimuli led to more severe oxidative stress and lipid peroxidation (Figures 6A–C) as compared with the PrP^106−126^ only treatment group. Moreover, deficiency in CL externalization also decreased intracellular ATP levels (Figure 6D) and mitochondrial membrane depolarization (Figures 6E, J) to a greater extent. Furthermore, we detected the activities of the respiratory chain complexes following PrP^106−126^ treatment. Neurotoxic peptides caused decreased activities of the four complexes to vary degrees (Figures 6F–I), and deficiency in CL externalization intensified these effects on complexes I and III. Thus, the inhibition of CL externalization to the outer mitochondrial membrane aggravates mitochondrial dysfunction induced by the PrP peptide.

## Discussion

Due to the non-regenerative nature of neurons, mitochondria have gradually become a central therapeutic target in neurodegenerative diseases due to their role in maintaining cell homeostasis (Hao et al., 2022; Lv et al., 2022). Here, we report that mitophagy is involved in the clearance of damaged mitochondria as an early response to PrP^106−126^ treated with N2a cells. This response was induced by CL translocation to the outer mitochondrial membrane, which stimulated LC3II-mediated autophagy of damaged mitochondria. Moreover, our data targeting PLS, NDPK-D, and CLS in a cellular model strongly support a vital role for CL translocation and repartition in PINK1 and DRP1 recruitment to the mitochondrial membrane. We further demonstrate that the mitochondrial dysfunction of different levels was intensified in PrP^106−126^ treatment following the prevention of CL externalization, indicating that the mechanisms and pathways of CL translocation may present new targets for drug discovery in prion disease.

Mitophagy is regarded as a regulator in several CNS disorders (Geisler et al., 2010b; Jin and Youle, 2012; Tang et al., 2016; Wang et al., 2019) including prion disease (Forloni et al., 2019). In accordance, we discovered dramatically increased mitophagy induced by PrP^106−126^ in N2a cells, which decreased for hours. Rescuing damaged mitophagy via PINK1/Parkin signaling, the main ubiquitination-mediated mitophagy pathway would alleviate apoptosis induced by PrP^106−126^ on N2a cells (Li et al., 2022). These data indicate that mitophagy may play a protective role against neuronal death prior to the induction of apoptosis. To better understand the process of mitophagy in N2a cells stimulated by PrP^106−126^, we carried out a time-course assessment of mitophagy at different levels. We found that mitophagy induced by PrP^106−126^ started at 1 h and peaked at 6 h after treatment with PrP^106−126^, and our previous studies using the TUNEL assay have demonstrated that cells gradually suffer cell death and cytochrome c release after 6 h (Song et al., 2014, 2017a,b; Li et al., 2018). This tendency may provide evidence that cells attempt to clear damaged mitochondria in order to circumvent the necessity to engage in apoptosis after exposure to the PrP^106−126^ peptide. Moreover, CL also plays a complex role in apoptosis, as previously reported (Kagan et al., 2009; Santucci et al., 2014; Mao et al., 2016; Sparvero et al., 2016); therefore, the potential effects and specific functions of CL in apoptosis induced by PrP^106−126^ require further investigation.

Furthermore, considering our previous studies on the function of the ubiquitination-mediated PINK1/Parkin pathway, we further determined its potential role in the induction of mitophagy following treatment with PrP^106−126^. Mitophagy activated by CL externalization has been found to be essential and unique due to the exclusive mitochondrial location of CL, during which externalized CL is rapidly sequestered by autophagosomes and directly binds to LC3II to initiate the recognition of injured mitochondria (Chu et al., 2013; Kagan et al., 2016). A recent study also demonstrated that CL displays different affinities for various members of the LC3 subfamily, which may play disparate roles in CL-mediated mitophagy (Iriondo et al., 2022); therefore, CL can also be regarded as a mitophagic receptor (Chu et al., 2013) that functions as a protective factor in cells, especially neurons. In traumatic brain injury, CL-mediated mitophagy acts as an endogenous neuroprotective process to eliminate damaged mitochondria (Chao et al., 2019). Meanwhile, we also discovered that the CL content was decreased after PrP^106−126^ peptide treatment via NAO, which specifically binds with high affinity to CL but not to zwitterionic or other anionic phospholipids. Similarly in Parkinson's disease (PD), several studies have reported that a general decrease in the CL content is also detected in different parts of the brain in both patients (Lloyd et al., 2019; Gilmozzi et al., 2020) and mice (Gao et al., 2017). Moreover, further research uncovered that α-Syn, the pathogenic factor of PD, can interact directly with CL to inhibit mitophagy and other cellular processes, which aggravates the progression of PD (Ryan et al., 2018). Several studies have also reported that the CL content displays a similar tendency in Alzheimer's disease (AD) (Guan et al., 1994; Monteiro-Cardoso et al., 2015; Kurokin et al., 2021). Although the potential function of CL in AD is still obscure, CL plays an essential role. Here, we show that non-ubiquitination-related CL-mediated mitophagy also plays a protective role in clearing injured mitochondria to maintain normal neuronal function after PrP^106−126^ is stimulated on N2a cells. Moreover, we measured the possible contribution of PINK1/Parkin to mitophagy as a result of CL repartition and discovered that PINK1 recruitment was decreased after treatment with PrP^106−126^ on N2a cells; however, there was no effect on Parkin. It has been reported that the ablation of ALCAT1, an acyltransferase that catalyzes the pathological remodeling of CL, significantly improves mitophagy in PD by promoting the recruitment of Parkin to dysfunctional mitochondria (Song et al., 2019). By contrast, another study found that tafazzin deficiency-induced decreases in the CL content have no significant influence on PINK1/Parkin (Hsu et al., 2015). In the present study, we demonstrate that CL promoted electron transport between ubiquinone and complex I in order to rescue PINK1 deficiency. Furthermore, considering that Parkin is involved in a complex mitophagic activation pathway and is responsible for directing the autophagic clearance of defective mitochondria, which is not only activated by PINK1-mediated phosphorylation but also by other ubiquitinated mitophagic signaling proteins, we suspect that the indifference of Parkin after inhibiting CL externalization may be due to signals other than PINK1 activation (Geisler et al., 2010a; Jin and Youle, 2012; Durcan and Fon, 2015; Seirafi et al., 2015). Therefore, the potential effects of CL externalization on PINK1/Parkin require further exploration.

Cardiolipin externalization can also influence DRP1 since CL provides GTPase activity and other complex functions for the activation of DRP1 in mitochondrial fission (Bustillo-Zabalbeitia et al., 2014; Francy et al., 2017; Kameoka et al., 2018; Mahajan et al., 2021). Here, confocal microscopy and immunoblotting results show that inhibiting CL translocation at its peak decreased DRP1 recruitment to the mitochondrial membrane. Our previous study found that PrP^106−126^ results in a gradual increase in DRP1 recruitment to mitochondria, but overactive DRP1 eventually causes mitochondrial fragmentation at both cellular and animal levels, indicating that DRP1 recruitment may play a dual role (Li et al., 2018). Our results indicate that the inhibition of CL externalization at its peak decreased DRP1 recruitment, which may influence damaged mitochondrial clearance through fission. It has been previously demonstrated that reduced CL externalization due to ablated ALCAT1 restores mitochondrial dynamics in both PD and coronary artery disease by preventing DRP1 function (Li et al., 2010; Song et al., 2019; Jia et al., 2021). More detailed experiments regarding the effect of CL externalization on DRP1 recruitment are required to explore the underlying mechanisms.

Our study also shows that the deficiency of CL-mediated mitophagy intensifies mitochondrial oxidative stress and dysfunction due to CL externalization. Considering that CL plays a role in the structural integrity and enzymatic activity of all complexes of the respiratory chain to produce ATP (Dudek et al., 2013, 2016), PrP^106−126^ peptide causes mitochondrial dysfunction and oxidative stress, which decreases mitochondrial ATP production. PrP^106−126^ peptide-induced externalization of CL from the inner to the outer mitochondrial membrane acts as a signal for the recruitment of LC3II and the initiation of mitophagy to clear damaged mitochondria. Inhibition of CL externalization aggravates mitochondrial oxidative stress and dysfunction, suggesting that the externalization of CL induced by PrP^106−126^ peptide plays a protective role against the accumulation of damaged mitochondria. Previous studies have demonstrated that CL functions in the respiratory chain to stabilize super-complex formation in the inner mitochondrial membrane (Fry and Green, 1981; Zhang et al., 2002; Arnarez et al., 2016). CL externalization leads to an imbalance in the cycle, which further triggers the dysfunction of different respiratory complexes in diverse diseases (Arias-Cartin et al., 2012; Schwall et al., 2012). Here, we found that the inhibition of CL externalization after PrP^106−126^ peptide treatment significantly intensified the dysfunction of complexes I and III, aggravating abnormalities in all the respiratory complexes. It has been highlighted that CL can modulate the structure and dynamics of respiratory complex I by specifically binding to certain subunits (Jussupow et al., 2019). Additionally, CL can also affect the function of complex III by preventing its combination with complex IV (Zhang et al., 2005). Moreover, another study showed that CL externalization to the outer mitochondrial membrane in PD can pull α-syn monomers out from preformed fibrils, facilitating their refolding, and effectively buffering synucleinopathy, which may explain the prion-like transmission of α-syn (Ryan et al., 2018). Whether there exists a similar mechanism of CL exposure to the outer mitochondrial membrane upon PrP^106−126^ protein refolding remains to be explored. The internal regulatory mechanism of CL externalization is complex, and several molecular signals have been uncovered (Kagan et al., 2014). CL translocation and repartition are regulated by SAM50, the depletion of which results in the externalization of CL and mitochondrial outer and inner membrane (including crista membrane) remodeling, triggering Bax mitochondrial recruitment, and mtDNA aggregation and release (Chen et al., 2022). The regulatory mechanism of CL externalization induced by prion disease still needs to be explored. Mitochondrial transplantation is gradually becoming an innovative treatment to cure several neurodegenerative diseases including PD and AD, displaying good efficacy in both cellular and animal models (McCully et al., 2017; Lim, 2020; Liu et al., 2021). Although there is still much work to be done until readiness for clinical application, the importance of mitochondrial homeostasis is recognized.

## Conclusion

**Figure 7:**
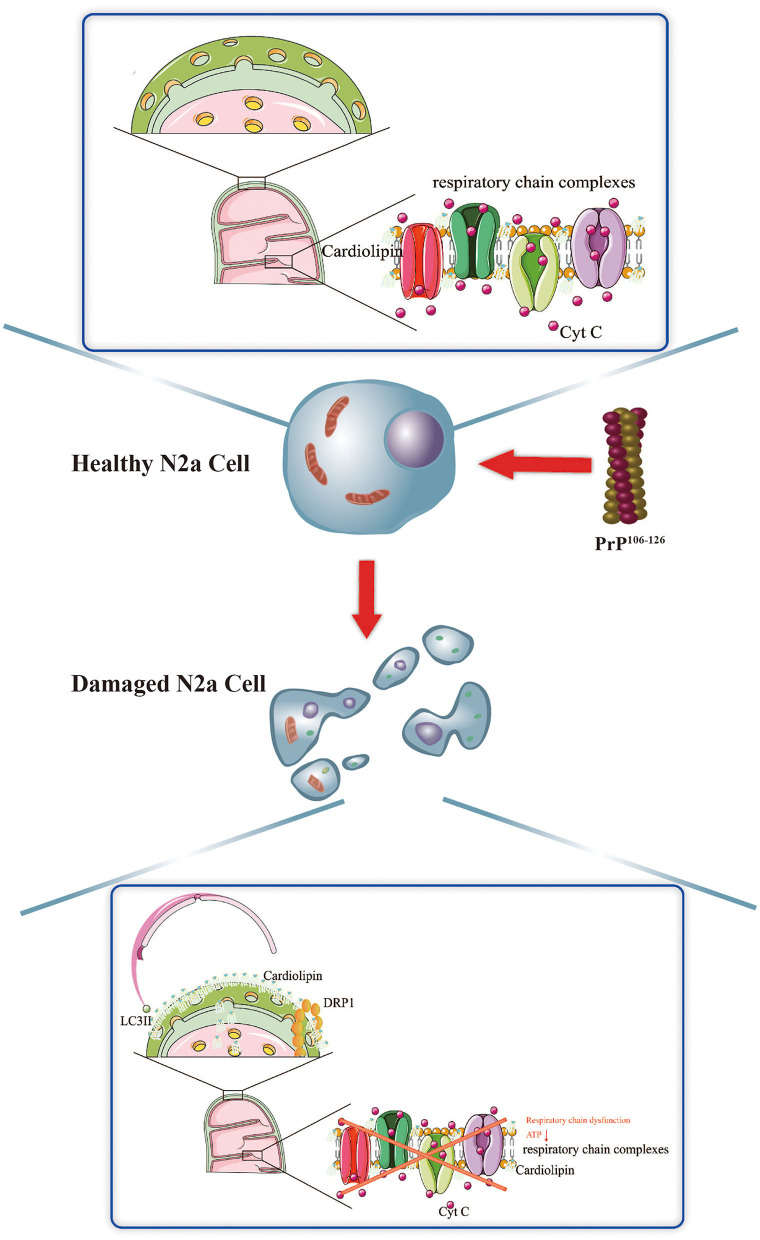
Schematic diagram summarizing the proposed role of CL in PrP^106−126^-associated mitophagy.

Taken together, we demonstrate that the activation of CL-mediated mitophagy, and the potential influence of CL translocation to the outer mitochondrial membrane on PINK1 and DRP1, is an essential response elicited by PrP^106−126^ stimuli on N2a cells. Suppression of CL-mediated mitophagy worsens the overall outcome of N2a cells stimulated by PrP^106−126^, likely via several different mechanisms. Our findings provide a better understanding of the potential role of mitophagy stimulated by PrP^106−126^ on N2a cells, in particular non–ubiquitination-mediated mitophagy, laying the foundation for the discovery of new small molecule regulators of mitophagy that could act as potential novel therapeutic targets, not only in prion disease but also in other CNS disorders (Figure 7).

## Data availability statement

The original contributions presented in the study are included in the article/Supplementary material, further inquiries can be directed to the corresponding author.

## Author contributions

Material preparation, data collection, and analysis were performed by DY and LY. The first draft of the manuscript was written by DY, and all other authors commented on previous versions of the manuscript. All authors contributed to the study's conception and design. All authors read and approved the final manuscript.
