# Supplementary material for: Cardiolipin externalization mediates prion protein (PrP) peptide 106–126-associated mitophagy and mitochondrial dysfunction
Source: Front Mol Neurosci. 2023 Jun 2;16:1163981. doi: 10.3389/fnmol.2023.1163981 (PMC10272765; doi:10.3389/fnmol.2023.1163981)
Supplement: Supplementary file 1 [file Data_Sheet_1.docx]

Supplementary material

To prove our concentration in our article reasonable and scientific, we detected the cell death data and cell apoptosis data using 150µM peptide on N2a cell after 6 hours and 24 hours and all procedures were strictly following the manufacturer’s instructions. The PrP^106-126^ scrambled sequence was: MEVGWYRSPFSRVVHLYRNGK referred from Benoit Schneider’s paper[1] and synthesized and purified by Sangon Biotech.

We used CCK8 (Beyotime) to detect the cell death. From the CCK8 data (in the figure1A), we observed that the cell viability was close to 85% and 55% after 150µM peptide treated on N2a cell at 6 hours and 24 hours. As to this PrP concentration, in Zhiping Li’s paper[2], she detected the N2a cell viability is nearly 50% under 150µM PrP^106-126^ condition for 24 hours using CCK8 (Figure 2C). And Wei Wu’s and Chaosi Li’s papers also demonstrated the similar result, whose cell viabilities are nearly 55% and closely to 50% separately under the same condition showing in the Figure 3Aand Figure 4A[3, 4]. Moreover, in several Zhiqi Song’s and Mengyu Lai’s papers, they also detected the cell viability of N2a was lower than 50% after 24 hours treatment with 200 µM PrP^106-126^[5-9]. Besides, several researches using 100µM PrP^106-126^ also demonstrated that N2a cell viability is between 55%-65%[10, 11]. Besides, In 2003, Tullio Florio used 100 µM PrP^106-126^ on cell to provide the Evidence that a soluble variant of the 106–126 peptide is neurotoxic[12]. And A CORSARO in 2006 also discussed the different concentrations of PrP^106-126^ from 10 µM to 100 µM and chose 100 µM to do the apoptotic experiments[13].

To comprehensively demonstrate the effect of 150µM PrP^106-126^ on N2a cellar apoptosis, we used FITC Annexin V apoptosis detection kit with PI (BD, 556547), TUNEL assay, and transmission electron microscopy (TEM) to detect the temporal course of apoptosis based on 150µM PrP^106-126^ treatment. From the data of FITC Annexin V apoptosis detection (figure 1B), it showed that 150µM PrP^106-126^ will lead to a temporal course of apoptosis and the apoptotic ratio was more than 60% after24 hours treatment, and the ratio of early apoptotic cells to late apoptotic cells is 16 in 24 hours treatment. Combined with the TEM data (figure 1C), it also showed that the typical apoptotic hallmarks in the nucleus, such as chromatin rearrangements, condensation or release into the cytoplasm, nuclear condensation, or fragmentation, were significantly appeared at PrP^106-126^ treatment for 24 hours. Moreover, the TUNEL assay (figure 1D) also displayed the similar trendy of apoptosis after 150µM PrP^106-126^ treated with N2a cells for 24 hours. These data were all displayed that 150µM PrP^106-126^ will cause the significant apoptosis of N2a cells after 24 hours treatment.

Moreover, we also detected the state of 150 µM PrP^106-126^ aggregation. Thioflavin T (Th T, MCE: HY-D0218) binding assay was produced according with these two papers[12, 14] by using Luminometer at 450/480 nm and confocal microscopy. From the data of Luminometer (figure 1E), we found that the Th T fluorescence was significant increased at 24 hours and there is no significant increase at 6 hours after Th T was treated with N2a cells. Moreover, we also directly detect the polymerized aggregation of 150 µM PrP^106-126^ solution showing in the figure 1F. According to Shahdat Hossain’s paper[15], 150 µM PrP^106-126^ displayed obviously polymerized. Besides, Figure 1G displayed the 150 µM PrP^106-126^ treated on N2a cells for 24 hours. It showed that the 150 µM PrP^106-126^ could significantly induce PrP protein aggregation referred from figure amyloid protein in Picken’s book[16] and Tao’s paper[17].

Therefore, we reckon that 150µM PrP^106-126^ treatment could better explore the effect of PrP^106-126^ on mitophagy in N2a cells, which can provide directions for the following aspects of research on prion diseases: (i) to find the molecular pathways that trigger the accumulation of damaged mitochondria in neurons, and (ii) to regulate mitophagy as a means for therapy.


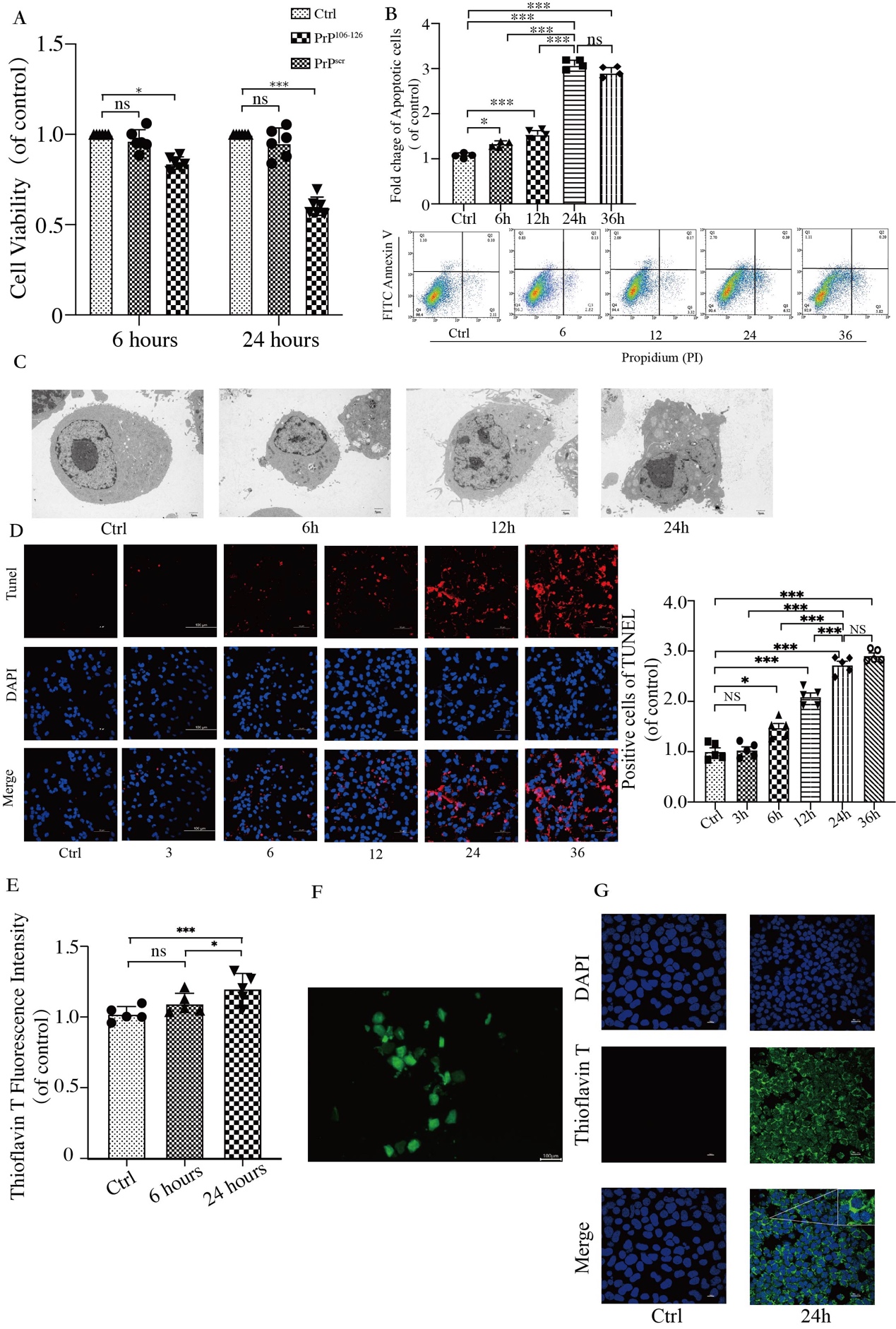


Figure 1 the toxicity data for treatment of cells with 150µM peptide and aggregation profiles. A. Cell viability of N2a cell treated with 150µM PrP^106-126^ after 6 and 24 hours. B. the temporal course of Annexin V-FITC Apoptosis assay for N2a cells treated with 150µM PrP^106-126^. C. the temporal course of N2a cellular apoptosis by TEM after 150µM PrP^106-126^ treatment, scale bar: 5 μm. D. the temporal course of TUNEL assay of N2a cells treated with 150µM PrP^106-126^, Scale bars: 50 μm.

E: Thioflavin T fluorescence assay of N2a cells treated with 150µM PrP^106-126^. F: Thioflavin T staining of polymerized amyloid aggregates of 150µM PrP^106-126^ solution. Scale bars: 100 μm. G. Thioflavin T staining of N2a cells treat with 150µM PrP^106-126^ for 24 hours. Scale bars: 100 μm. Data are presented as the mean ±SD; ns, not significant; *P < 0.05; **P < 0.01; ***P < 0.001. All experiments were repeated at least three times and PrP106–126 treatment was 6 hours, causing a peak in mitophagy. Statistical analysis was done using 1-way analysis of variance with Tukey posttests and unpaired t test (2 tailed).
